# Supplementary material for: A co-produced review of the experiences of Black male detention under mental health legislation: Challenging discrimination in psychiatry using The Silences Framework
Source: PLOS Ment Health. 2025 Apr 9;2(4):e0000041. doi: 10.1371/journal.pmen.0000041 (PMC12798175; doi:10.1371/journal.pmen.0000041)
Supplement: S1 Appendix — (DOCX) [file pmen.0000041.s001.docx]

Initial searches

| **EBSCO** |  |
| --- | --- |
| (( “Mental Health” OR “mental illness” OR “mental stability” OR “mental disorder” OR “emotional disorder” )) AND (( “black men” OR BAME OR “black man” OR “black person” OR “black adult male” )) AND (Detention OR detained OR incarcerated) | 48 |
| (( “Mental Health” OR “mental illness” OR “mental stability” OR “mental balance” OR “mental disorder” OR depression OR “mental sickness” OR “emotional disorder” )) AND (( “black men” OR BAME OR “black man” OR “black person” OR “black adult male” )) AND (Detention OR detained OR incarcerated) | 48 |
| (( “Mental Health” OR “mental illness” OR “mental stability” OR “mental balance” OR “mental disorder” OR depression OR “mental sickness” OR “emotional disorder” )) AND (( “black men” OR BAME OR “black man” OR “black person” OR “black adult male” )) AND (( “police detention” OR detention )) | 7 |
| (( “black men” OR BAME OR “black man” OR ethnicity OR “black adult male”  )) AND (( “Mental Health” OR “mental illness” OR “mental disorder” OR depression OR “mental sickness” OR “emotional disorder” )) AND (( Detention OR “involuntary hospitalisation” )) | 148 |
| (( "black men" OR "black males" OR "african american men" )) AND (( “Mental Health” OR “mental illness” OR “mental disorder” OR depression OR “mental sickness” OR “emotional disorder” )) AND (( Detention OR “involuntary hospitalisation” )) | 20 |
| (( “Mental Health” OR “mental illness” OR “mental stability” OR “mental balance” OR “mental disorder” OR depression OR “mental sickness” OR “emotional disorder” )) AND (( “black men” OR BAME OR “black man” OR ethnicity OR “black adult male” )) AND (( Detention OR “involuntary hospitalisation” )) | 148 |
| (( “Mental Health” OR “mental illness” OR “mental stability” OR “mental balance” OR “mental disorder” OR depression OR “mental sickness” OR “emotional disorder” )) AND (( “black men” OR “black man” OR “black adult male” ) ) AND (( Detention OR “involuntary hospitalisation” or "involuntary hospitalization")) | 6 |
| (( "black men" OR "african american men" OR "black males" )) AND (( "mental health" OR "mental illness" OR "mental disorder" OR "psychiatric illness" )) AND (( Detention OR "involuntary hospitalisation" OR “involuntary hospitalization”)) | 10 |
| (((black OR african OR afri OR caribbean OR BME OR BAME OR minority OR ethnic OR ethnicity OR race OR racist OR racial) AND ("mental health" OR "mental health act" OR psychiatry OR psychiatric)) AND (detention OR detain OR custody OR incarceration OR incarcerated OR compulsory OR sectioned OR "Section 136" OR "Section 135" OR "Section 2" OR "Section 3")) | 3,081 |
| (((((black) OR (BME)) OR (afri*)) AND (mental health)) AND (hospital)) AND (detention) | 61 |
| (((((BME) OR (minority groups)) AND (black)) AND (mental health act)) AND (detention)) AND (hospital) | 5 |
| Black AND male AND (“mental health” or “mental illness” or “mental disorder” or “psychiatric illness”) AND (Detention OR Incarceration OR Hospital OR Section*) | 3,995 |
| Black AND male AND (“mental health” or “mental illness” or “mental disorder” or psychiatric illness”) AND (Detention OR Incarceration OR Hospital OR Sectioned) | 1,929 |
| **Proquest** |  |
| Ab(( “Mental Health” OR “mental illness” OR “mental stability” OR “mental disorder” OR “emotional disorder” )) AND (( “black men” OR BAME OR “black man” OR “black person” OR “black adult male” )) AND Ab (Detention OR detained OR incarcerated) | 114 |
| Ab(( “Mental Health” OR “mental illness” OR “mental stability” OR “mental balance” OR “mental disorder” OR depression OR “mental sickness” OR “emotional disorder” )) AND (( “black men” OR BAME OR “black man” OR “black person” OR “black adult male” )) AND Ab (Detention OR detained OR incarcerated) | 129 |
| Ab(( “Mental Health” OR “mental illness” OR “mental stability” OR “mental balance” OR “mental disorder” OR depression OR “mental sickness” OR “emotional disorder” )) AND (( “black men” OR BAME OR “black man” OR “black person” OR “black adult male” )) AND Ab(( “police detention” OR detention )) | 22 |
| (( “black men” OR BAME OR “black man” OR ethnicity OR “black adult male”  )) AND Ab (( “Mental Health” OR “mental illness” OR “mental disorder” OR depression OR “mental sickness” OR “emotional disorder” )) AND Ab (( Detention OR “involuntary hospitalisation” )) | 310 |
| (( "black men" OR "black males" OR "african american men" )) AND Ab (( “Mental Health” OR “mental illness” OR “mental disorder” OR depression OR “mental sickness” OR “emotional disorder” )) AND Ab (( Detention OR “involuntary hospitalisation” )) | 36 |
| (( “Mental Health” OR “mental illness” OR “mental stability” OR “mental balance” OR “mental disorder” OR depression OR “mental sickness” OR “emotional disorder” )) AND (( “black men” OR BAME OR “black man” OR ethnicity OR “black adult male” )) AND (( Detention OR “involuntary hospitalisation” )) | 207 |
| Ab(( “Mental Health” OR “mental illness” OR “mental stability” OR “mental balance” OR “mental disorder” OR depression OR “mental sickness” OR “emotional disorder” )) AND (( “black men” OR “black man” OR “black adult male” ) ) AND Ab(( Detention OR “involuntary hospitalisation” or "involuntary hospitalization")) | 21 |
| (( "black men" OR "african american men" OR "black males" )) AND Ab(( "mental health" OR "mental illness" OR "mental disorder" OR "psychiatric illness" )) AND Ab(( Detention OR "involuntary hospitalisation" OR “involuntary hospitalization”)) | 31 |
| (black OR african OR afro OR caribbean OR BME OR BAME OR minority OR ethnic OR ethnicity OR race OR racist OR racial) AND ("mental health" OR "mental health act" OR psychiatry OR psychiatric) AND (detention OR detain OR custody OR incarceration OR incarcerated OR compulsory OR sectioned OR "Section 136" OR "Section 135" OR "Section 2" OR "Section 3") | 2,082 |
| (((((black) OR (BME)) OR (afri*)) AND Ab(mental health)) AND (hospital)) AND (detention) | 26 |
| (((((BME) OR (minority groups)) AND (black)) AND Ab(mental health act)) AND (detention)) AND (hospital) | 5 |
| Black AND male AND Ab (“mental health” or “mental illness” or “mental disorder” or “psychiatric illness”) AND Ab(Detention OR Incarceration OR Hospital OR Section*) | 3,296 |
| Black AND male AND Ab(“mental health” or “mental illness” or “mental disorder” or psychiatric illness”) AND Ab(Detention OR Incarceration OR Hospital OR Sectioned) | 1704 |
| **PMC Pubmed Europe** |  |
| (( “Mental Health” OR “mental illness” OR “mental stability” OR “mental disorder” OR “emotional disorder” )) AND (( “black men” OR BAME OR “black man” OR “black person” OR “black adult male” )) AND (Detention OR detained OR incarcerated) | 475 |
| (( “Mental Health” OR “mental illness” OR “mental stability” OR “mental balance” OR “mental disorder” OR depression OR “mental sickness” OR “emotional disorder” )) AND (( “black men” OR BAME OR “black man” OR “black person” OR “black adult male” )) AND (Detention OR detained OR incarcerated) | 524 |
| (( “Mental Health” OR “mental illness” OR “mental stability” OR “mental balance” OR “mental disorder” OR depression OR “mental sickness” OR “emotional disorder” )) AND (( “black men” OR BAME OR “black man” OR “black person” OR “black adult male” )) AND (( “police detention” OR detention )) | 158 |
| (( “black men” OR BAME OR “black man” OR ethnicity OR “black adult male”  )) AND (( “Mental Health” OR “mental illness” OR “mental disorder” OR depression OR “mental sickness” OR “emotional disorder” )) AND (( Detention OR “involuntary hospitalisation” )) | 1,554 |
| (( "black men" OR "black males" OR "african american men" )) AND (( “Mental Health” OR “mental illness” OR “mental disorder” OR depression OR “mental sickness” OR “emotional disorder” )) AND (( Detention OR “involuntary hospitalisation” )) | 196 |
| (( “Mental Health” OR “mental illness” OR “mental stability” OR “mental balance” OR “mental disorder” OR depression OR “mental sickness” OR “emotional disorder” )) AND (( “black men” OR BAME OR “black man” OR ethnicity OR “black adult male” )) AND (( Detention OR “involuntary hospitalisation” )) | 1,554 |
| (( “Mental Health” OR “mental illness” OR “mental stability” OR “mental balance” OR “mental disorder” OR depression OR “mental sickness” OR “emotional disorder” )) AND (( “black men” OR “black man” OR “black adult male” ) ) AND (( Detention OR “involuntary hospitalisation” or "involuntary hospitalization")) | 131 |
| (( "black men" OR "african american men" OR "black males" )) AND (( "mental health" OR "mental illness" OR "mental disorder" OR "psychiatric illness" )) AND (( Detention OR "involuntary hospitalisation" OR “involuntary hospitalization”)) | 180 |
| ("black" OR "african" OR "caribbean" OR "BME" OR "BAME" OR "minority" OR "ethnic*" OR "race") AND ("mental health act" OR "psychiatr*") AND ("detention" OR "detained" OR "incarcerat*" OR "compulsory" OR "section*") AND (FIRST_PDATE:[1983 TO 2023]) | 2,414 |
| (((((black) OR (BME)) OR (afri*)) AND (mental health)) AND (hospital)) AND (detention) | 1,352 |
| (((((BME) OR (minority groups)) AND (black)) AND (mental health act)) AND (detention)) AND (hospital) | 288 |
| Black AND male AND (“mental health” or “mental illness” or “mental disorder” or “psychiatric illness”) AND (Detention OR Incarceration OR Hospital OR Section*) | 228 |
| Black AND male AND (“mental health” or “mental illness” or “mental disorder” or psychiatric illness”) AND (Detention OR Incarceration OR Hospital OR Sectioned) | 228 (same as above) |
