# Supplementary material for: A co-produced review of the experiences of Black male detention under mental health legislation: Challenging discrimination in psychiatry using The Silences Framework
Source: PLOS Ment Health. 2025 Apr 9;2(4):e0000041. doi: 10.1371/journal.pmen.0000041 (PMC12798175; doi:10.1371/journal.pmen.0000041)
Supplement: S2 Appendix — (DOCX) [file pmen.0000041.s002.docx]

| **Score** | **Ethnicity Categorisation** | **Gender** | **Detention** |
| --- | --- | --- | --- |
| **2** | Inappropriate ethnic groups or difficult to ascertain ethnicity. Third party categorisation, unclear. | Does not differentiate between genders | Admittance to a mental health ward/ unit/ other without specific information on compulsory detention under mental health legislation |
| **1** | BAME or BME not separated out, groups lumped together. Self-reported needs interpretation (i.e. States Black Zimbabwean and interpreted as Black African to comply with UK Gov guidelines) | Differentiates between gender | States mental health detention |
| **0** | Clear definition for Black, Black African and or Caribbean, Black mixed or Black other/ unspecified. UK Government defined categories. | Differentiates between gender and links specifically to ethnic identity, specifically Black men. | States mental health detention under legislation |

Ethnicity, gender, detention score
