# Supplementary material for: A co-produced review of the experiences of Black male detention under mental health legislation: Challenging discrimination in psychiatry using The Silences Framework
Source: PLOS Ment Health. 2025 Apr 9;2(4):e0000041. doi: 10.1371/journal.pmen.0000041 (PMC12798175; doi:10.1371/journal.pmen.0000041)
Supplement: S3 Appendix — (DOCX) [file pmen.0000041.s003.docx]

*Table of initial Themes and Subthemes*

| Themes | Sub-themes |
| --- | --- |
| Environment and Practise;  This theme explores practise within mental health services. | - Oppressive practice - Poor practise - Violent practise - Excessive prescribed drugs - Wrong medication - Black patients are treated as inferior - The care given to Black patients is poor - Why do Black patients present with ‘unusual behaviour? - Racial abuse from staff and patients - Data protection - Rules and regimes - Quality of training and practise - More complex pathways for Black men - Black men more likely to be in medium and high secure services - Over representation of police detention - Cultural appropriate treatment - Diet and food as part of holistic treatment - Professional teams are not curious about patients - Lack of empathy - Bias again Black people – preconceived ideas - Psychiatric abuse - Does the individual agree with their diagnosis? - Challenging your care |
| Identity:  This theme highlights how different idenfities, on top of race, can influence treatment in and experiences of services, and explores how identity is presented within research. | - Black men more likely to be younger and have longer hospitalisations - Black people are seen as a monolith and defined as one category - Colourism, Black people treated differently depending on how light or deep their skintone is - Class - Neurodiversity - Physicality - Culture and traditions - Spirituality - Religion - Family |
| Language and Communication:  This theme explored how language used by professionals and a lack of communication between them and patients and carers contribute to poor experiences of mental health services. | - Clinical gaslighting - Intersectionality - Language barriers - Human rights and international law - Family’s role in care - Advocates and support with no family - Understanding the diagnosis - Accountability |
| Power and First Order Practise and Profesisonal Curiosity:  This theme explored how power held by the professional and by mental health services supercede patients and carers and the preference for first order, blanket rules over person centred, case-by-case decision making. | - Elitism - Class - Hierarchy - Patient led and patient consulted care - Lack of insight into care - Patients being criminalised - Under-diagnosis of non-psychotic disorders - Over diagnosis of psychotic disorders - Element of ‘working out’missing from services, lack of curiosity about the whole person - Separating the person and their mental illness as they are not one and the same |
