# Supplementary material for: A co-produced review of the experiences of Black male detention under mental health legislation: Challenging discrimination in psychiatry using The Silences Framework
Source: PLOS Ment Health. 2025 Apr 9;2(4):e0000041. doi: 10.1371/journal.pmen.0000041 (PMC12798175; doi:10.1371/journal.pmen.0000041)
Supplement: Attachment — Submitted filename: Response to Reviewers.docx [file pmen.0000041.s004.docx]

Response to Reviewers

Thank you for giving us the opportunity to resubmit this paper now it has been updated, as per your request. Our original submission PMEN-D-23-00059 was stated to be out of date as our original searches were completed over a year prior to submission. We have completed a full further search using the same search terms, and have produced another PRISMA diagram to address this additional search. The final included papers have been integrated into the findings and discussion section.

Two papers were added from our search, and additional information from these papers was added to the findings. The papers were useful in adding to the arguments that we had previously made.
