# Supplementary material for: A co-produced review of the experiences of Black male detention under mental health legislation: Challenging discrimination in psychiatry using The Silences Framework
Source: PLOS Ment Health. 2025 Apr 9;2(4):e0000041. doi: 10.1371/journal.pmen.0000041 (PMC12798175; doi:10.1371/journal.pmen.0000041)
Supplement: Attachment — Submitted filename: Responses to reviewers.docx [file pmen.0000041.s005.docx]

|  | Comment | Response |
| --- | --- | --- |
| **Reviewer 1** |  | We were very grateful to both reviewers for their comprehensive reviews. We felt that both challenged us, and their comments have made this paper much stronger as a result. |
| Introduction |  |  |
| 1 | In the abstract, you mention 34 papers, and in the second line of your intro you state 32 papers. I may be confused that these are high quality papers and the other two were not? | Complete - there were 34 papers, this was an error. |
| 2 | The first paragraph of the introduction sounds like a repeat of the abstract. While summarising is useful, consider restructuring to avoid repetition. You might start with a global perspective, narrow down to Black African Caribbean individuals, and then focus on Black males specifically. | Complete - this now begins with the Global North, moving to England then Black men |
| 3 | The phrase “Of particular concern was the over-representation of Black African Caribbean people under the MHA” could benefit from a reference | Completed - Reference added |
| 4 | Ensure consistent capitalisation of terms like "Black" and "white" for clarity and consistency for example you state: “Black African Caribbean men are more likely to encounter the mental health system via the criminal justice route than their white counterparts”. Same as the proceeding sentence: “The most recent figures show that 65.3 per 100,000 of Black people were detained under section 136 compared to 29.1 per 100,000 in those of white ethnicity” and this sentence “Black people detained under the MHA in this year, in comparison to the reducing 74.7 per 100,000 white people” | Completed - Capitalisation is used for both throughout |
| 5 | When you introduce the Silences Framework, it feels like part Methods section and part Discussion section. I would suggest most of the discussion elements be left to the discussion section. | Completed. This has been shortened and moved to the methods section. |
| 6 | NHS - in a UK context people may know this, however, in a global context may be useful when using it first time to use the full “National Health Service”? | Completed - Changed to National Health Service (NHS) when used in the first incidence |
| Methods |  |  |
| 7 | May you give an explanation as to how you came to a thematic approach than focusing on the quantitative data. | Thank you for pointing this out, we believe that exploring experiences of people is explicitly qualitative. We have tried to make this clearer throughout the methods and our additions as a result of other comments in this review (detailed within this table) should help with strengthening this. |
| 8 | In the second paragraph of the Methods section, is the use of 'OR' here meant to indicate criteria rather than explicit Boolean operators? Using uppercase 'OR' might make it seem as though Boolean operators are being explicitly applied in a search strategy, which could be confusing if that is not the intention. | Completed - the addition of the words (using Boolean terms) before the sentence should ensure that it is clear. |
| 9 | May you provide a reference and description at first mention of “Covidence”? | Completed - reference added. |
| 10 | There are some grammatical issues, such as ensuring subject-verb agreement. For instance, “the initial search criteria was” should be “the initial search criteria were.” Reviewing for grammatical errors and shortening some sentences could improve readability. | Completed |
| 11 | Just to comment I enjoyed reading this bit. Well done: Additionally, two reviewers conducted an equality appraisal based on a previous version by Bhui et al., providing a score on how each included paper describes ethnicity, gender, and detention. This equality appraisal is included to showcase the differences in the explanations of these demographics and therefore the difficulties involved in comparing the data. As this is not an approved measurement tool, we did not use it to exclude any papers, but felt it was an important to include how vastly different demographics are recorded in healthcare, and how difficult that makes it for researchers to use the data to state anything with any certainty about equality, diversity, and inclusion issues. | Thank you! |
| Results |  |  |
| 12 | Clarification is needed on the number and purpose of workshops mentioned. Distinguishing between different workshops or summarising the results more concisely could reduce redundancy | Thank you for your comment. We have considered this at length, and decided that we do not want to reduce this information. Co-production should be about collaboration and we think it's important for other researchers to have a good understanding of what we did. We have put a summary sentence of how many workshops were run altogether. Page 9: We held a total of four workshops in various guises.  We also added some information about our approach at the beginning of the method: Co-producing a systematic review including people with lived experience is a novel approach, and there is currently no framework that determines how this is conducted or written for publication. Therefore, we have written openly about the way our research was conducted and have included as much explanation as possible in the hope that moving forward, a framework for co-producing research may be designed. |
| 13 | The results section focuses heavily on quantitative data despite your qualitative approach in the methods. Aligning these sections more closely could enhance coherence. | Completed: Whilst the exploration of experiences predominately requires a qualitative answer, we have synthesised the quantitative and qualitative data to provide a more holistic overview. Quantitative data was thematised by using the subheadings to the data commentary, much in the same way that qualitative data uses subheadings to describe themes. |
| 14 | Stigma plays a significant role in mental health experiences. However, in your manuscript, it receives an almost footnote explanation without sufficient wider context. You briefly mention stigma and then quickly move on to state that identity is complex and multifaceted. This transition feels abrupt, and it’s challenging to understand the full impact of stigma on the complex identities of Black African Caribbean men in this context. A more detailed examination of how stigma intersects with and exacerbates the challenges of these multifaceted identities would strengthen your analysis. For instance, how does stigma related to mental health interact with other forms of stigma (e.g., racial or socio-economic) faced by Black men? Providing a more integrated discussion could offer deeper insights into their experiences | Completed - a paragraph has been added on page 23 and 24 to clarify and strengthen this point: The primary concern raised by many Black men in the literature and from people with lived experience, is about their specific experiences of *racial discrimination*. However, there is a need to also emphasise the importance of intersectionality while acknowledging that race-specific issues remain a crucial part of the narrative. It is critical to clarify that Black men are most likely to be detained and suffer biases due to their Blackness; highlighting race as a primary factor and that these inequalities exist regardless of the intersectional identities.  This could be misinterpreted as a philosophical dilemma within the context of Black men’s multiple intersectionality's. Blackness is a primary inequality driver but, it should not be so reductionistic that it is seen in isolation from other intersecting identities such as gender, sexuality, class, and disability. Intersectionality theory highlights the double disadvantage and inequality that is experienced by people with mental illness who belong to multiple stigmatized social groups^55,56,57^.  Interventions aiming to reduce these inequalities need to be flexible and targeted towards intersectionality's (gender, sexuality, etc) rather than universal (Black men) to effectively address systemic inequality. Therefore, the desire to be seen as more than monolithic is not to take away the Black identity from the man but from a desire to be seen as more than a negative stereotype and re-address the stigma, and trauma rooted within this. Addressing these negative perceptions and racial biases is an important step toward reducing predetermined projections of mental health behaviours on to Black men during detention. Creating environments where people feel as though they are safe, they have a voice, and they are listened to will ensure that there is a reduction in fear, frustration and readmission ^48,52,53,54^. |
| 15 | You mention, “Intersectional identities need to be understood to address the holistic issues experienced by Black men, and it must be assumed that the individual may have experienced all these contextual factors, or none of them, with a wide spectrum of experiences in between.” This is followed by, “This supports the view that people should be treated as individuals and support should be tailored to their needs based on an understanding of culture, societal, and health needs.” Are these two statements related to specific literature or results from your review? If so, it would be beneficial to explicitly link these statements to your findings or to existing studies. Currently, it feels like the first statement is a general comment about intersectionality, while the second is a related but separate point about individualised treatment. Clarifying how these points are supported by your results or the literature would strengthen your argument. For example, you could reference specific studies that highlight the importance of considering intersectional identities in mental health treatment and how this informs the need for tailored support. | Completed: References added. The additional paragraph in the discussion also links to this. |
| 16 | You state, “Currently, the literature reports on Black people’s experiences only through the lens of their Blackness, a reductionist approach that fails to recognise the complexities of their self-identity; as such, biases against gender, sexuality, and class are not explored in tandem with race in much detail or beyond statistics.” However, isn't the stated aim of your review to address these very complexities and intersections? You begin by stating that Black people/men are most likely to be detained and suffer biases due to their Blackness. This seems to highlight race as a primary factor. Within the context of your intersecting concepts, it appears that you may be arguing against the reductionist view while simultaneously emphasising the significance of Blackness. This could be seen as a philosophical dilemma, but clarifying your stance could strengthen your argument. Are you suggesting that while Blackness is a significant factor, it should not be considered in isolation from other intersecting identities such as gender, sexuality, and class? Explicitly addressing this potential ambiguity and providing balanced explanations or examples from your findings could help resolve this apparent contradiction. | Completed - a paragraph has been added to clarify and strengthen this point |
| 17 | You state, “Seeking to understand the racialised experience in a vacuum can contribute to a cycle of silencing by not considering the narratives of those who have multiple marginalised identities.” While I agree with this statement, isn't there a risk of ignoring the significant inequalities faced by Black people, as highlighted in the earlier literature you included? These inequalities exist regardless of the intersectional identities. The primary concern raised by many Black individuals is that their specific experiences of racial discrimination are often overlooked in favour of other characteristics, which, while important, might dilute the focus on race-specific issues. This could make your study appear similar to the studies you were initially critical of, which tended to overlook the Black experience. It would be beneficial to balance your discussion by emphasising the importance of intersectionality while also acknowledging that race-specific issues remain a crucial part of the narrative. Providing clear examples and explanations of how both perspectives are integrated into your findings could enhance the depth and originality of your study. | Completed - a paragraph has been added to clarify and strengthen this point (as above) |
| 18 | The example you give, “In an example provided by an expert by experience, they explain that not only are they Black, but they are also a product of where they have been brought up, their sexuality, and much more. Those identities matter and will not only shape one’s own behaviour and perspective but will also most likely shape the behaviour and perspective of those who provide mental health care, similar to the ways Blackness can be perceived in services,” raises an important point. However, it could be interpreted as suggesting we should ignore their Blackness and focus on everything else. This is problematic because, as you acknowledge, the systemic issues faced by Black individuals often mean that their race is a primary factor in how they are treated, regardless of other intersecting identities. This example might be more appropriate in the discussion section where you could expand on this insight. You could explore how both the individual's broader identity and their race intersect to impact their experiences in the mental health system. This would allow you to provide a more detailed discussion on how systems need to consider both race and other intersecting identities to provide holistic and effective care. This is related to the two points above. I am not trying to silence their voice but it may be better amplified if it supports the narrative that you are trying to un-silence the voices of the Black experience. | Completed - a paragraph has been added to clarify and strengthen this point (as above) |
| 19 | I think your results section would benefit from the inclusion of quotes from the studies. Direct quotes can provide powerful, illustrative examples that bring the data to life and add depth to your analysis. They can help to highlight key findings and give voice to the participants’ experiences, making the results more engaging and impactful. Including quotes can also enhance the credibility of your findings by providing concrete evidence from the literature. | Due to the word count, we felt that this was not useful as people can go to the review papers themselves, but we will be including more quotes in our subsequent papers and the final NIHR report. |
| 20 | I suggest reviewing and clearly specifying the type of thematic analysis you are utilizing. Based on my understanding of your results, it seems you may have attempted to use reflexive thematic analysis. However, the results section contains a lot more discussion than actual analysis. It would be beneficial to ensure that the results section focuses on presenting the data and findings derived from the thematic analysis. More detailed analysis of the themes, supported by quotes and examples from the studies, would strengthen this section. Clarifying the methodological approach and ensuring a clear distinction between results and discussion will enhance the rigor and clarity of your manuscript. | This is already available: Analysis of the papers was through thematic analysis^22^, and was conducted through hybrid (online and in-person) workshops. Two people read each paper and extracted the main findings. Flexibility of thematic analysis allows for the generation of themes across various methods of research and across many different positionalities and theoretical epistemologies. The team read through a portion of the papers each before coming together to discuss, with data extraction in the form of collating themes from each paper and determining which were the ones that came up the most. The second workshop involved the whole research team and produced 50 subthemes with four potential overarching themes and Silences found in the literature. The third workshop included two researchers and two people with lived experience from the review team, with people with lived experience leading on reducing the 4 overarching themes to three, alongside articulating the corresponding Silences. |
| 21 | You state, “The perception that Black people all share the same views and experiences of mental health services contributes to the misunderstanding of their experiences. This contributes to a cycle of silencing experiences that do not align with more accepted or well-known experiences and leads to reductionist models that silence and disempower the racialised lived experience in research, mental health policy, and practice. Though there is a collective solidarity among Black communities in the UK, there is also intra-communal segregation between African and Caribbean communities, even some division within African and Caribbean communities based on country, island, and religion.” These claims and statements require references to support them or at least a disclaimer indicating that these are your views. While it is understood that there are differences and similarities in experiences, my understanding is that your study has primarily focused on comparisons. You have emphasized that "not all Black people are the same," but in doing so, you may have overlooked the fact that Black individuals are more likely to suffer from the inequalities you mention. It is important to balance your discussion by acknowledging these pervasive inequalities while exploring intra-communal differences. Providing references or explicitly stating that these are your interpretations will enhance the credibility and clarity of your argument | Completed: Added in two places: Some of the references to their words are left intentionally ambiguous as we have a responsibility to protect our colleagues and co-authors. / These have been presented as a discussion of the systematic review findings, before voicing the silences relating to those points. We believe that this approach helps in understanding what is not being said in current research, and it is important that each theme has a response by people with lived experience. The exploration of the themes is therefore set out as information from the existing literature, as analysed by the full team, with a discussion of the silences that people with lived experience feel is missing. |
| 22 | Ensure consistent capitalisation of terms like "Black" and "white" for clarity and consistency for example you state: “Black African Caribbean men are more likely to encounter the mental health system via the criminal justice route than their white counte | Completed - Capitalisation is used for both throughout |
| 23 | Your statement, “Bias, whether conscious or unconscious, is affecting the lives of Black men and means that psychiatric services are not considering cultural factors when interpreting symptoms or implementing care plans,” needs further clarification. Are you indicating that psychiatric services are not considering cultural factors, racial factors, or both? The sentence as currently written seems to conflate culture and race. It is important to clearly distinguish between these two concepts to avoid confusion. If you are referring to both cultural and racial factors, consider revising the sentence to explicitly state this. For example, “Bias, whether conscious or unconscious, is affecting the lives of Black men and means that psychiatric services are not considering either cultural or racial factors when interpreting symptoms or implementing care plans. | Completed - Ammended as suggested to “Bias, whether conscious or unconscious, is affecting the lives of Black men and means that psychiatric services are not considering either cultural or racial factors when interpreting symptoms or implementing care plans." |
| Discussion |  |  |
| 24 | You state, “Their voices are missing from literature until now, but the statistics show that the over-representation of Black men in mental health detention comes at a human and financial cost and demands attention.” Could you please clarify where these voices are explicitly included in the review? It would be beneficial to specifically indicate whose voice or view is being represented. I recall only one instance where a Black individual expressed wanting to be viewed beyond their Blackness, including their sexuality and upbringing. In the identified literature, it is not clear if these perspectives are from clinicians, researchers, or Black men themselves. Providing clear attribution of these views would enhance the clarity and impact of your discussion. For example, explicitly stating whether the perspectives are those of the Black men affected, the clinicians treating them, or the researchers studying them would provide a clearer understanding of the different viewpoints being presented | The Lived Experience group discussed this, and it was determined that we needed to be considerate to our named authors with lived experience. They can be vulnerable and so we have been deliberately ambiguous to protect them as much as possible. Added to the statement quoted is now: Their voices are missing from literature until now, and our unique Silences Framework systematic review has allowed for people with lived experience to voice their unheard experiences. The statistics show that the over-representation of Black men in mental health detention comes at a human and financial cost demanding attention ^19.^ Our findings add further meaning to this. |
| 25 | You state, “By ignoring the intersectionaities (CHECK SPELLING) that exist for Black men, there is a chance that misdiagnosis and mistreatment may occur, which is where racism, difficulties with staff and understanding the treatment given, and a lack of choice for treatment and support continues to be perpetuated.” I agree with this statement. Ignoring the existing racism may indeed lead to difficulties in staff providing appropriate care, support, and understanding of the treatment given. It would be beneficial to integrate this point with your earlier statements in the results section to provide a cohesive argument. I appreciate your subsequent statement, “Addressing perceptions and racial bias is an important step toward reducing predetermined projections of mental health behaviours on to Black men during detention.” Thank you for acknowledging the presence of racial bias. Ensuring that both intersectionalities and the direct impact of racism are addressed will strengthen your argument and provide a more comprehensive understanding of the issues at hand | Thank you for the comment, this has been addressed through comments 16-18 above. Spelling has been changed. |
| 26 | You state, “The studies included in the systematic review were subject to a quality review to minimize the number due to the time afforded through the funding of this part of the wider project. This in itself is a form of silencing, and we did not include any grey literature.” I appreciate your honesty about this limitation. However, I did not get a clear reason as to why grey literature was explicitly excluded. Providing a rationale for this exclusion would help readers understand the scope and limitations of your review more fully. For example, you could explain whether it was due to concerns about the quality and reliability of grey literature, resource constraints, or other factors. This additional context would enhance the transparency and comprehensiveness of your methodology | Completed - The method section shows that grey literature was included in the search. The discussion section explains "The studies included in the systematic review were subject to a quality review to minimise the number due to the time afforded through the funding of this part of the wider project. This in itself is a form of silencing. During the quality appraisal all grey literature was excluded." |
| 27 | One of your recommendations is “support for carers.” However, this aspect was not addressed during your review, either through the identified studies or through the voice of a carer in your working group. Including the perspectives and experiences of carers would strengthen this recommendation and provide a more comprehensive understanding of the support needed. It would be beneficial to integrate findings related to carer experiences or to highlight the lack of such perspectives in the current literature as a limitation that needs to be addressed. This will add depth to your recommendation and ensure that the voices of carers are adequately represented in your review. Which currently seem to be silenced. | We have taken this out and it will feature more prominently in the findings paper. |
| 28 | You state, “Our research found that patients want to be actively involved in the decision-making regarding their treatment.” However, I did not see this mentioned elsewhere in the review. For consistency and thoroughness, it would be beneficial to ensure that this important finding is clearly presented and supported by data within the main body of your review. If this was indeed a finding from your research, please provide details on how this conclusion was reached and reference any relevant data or quotes from the studies. This will help to substantiate your statement and enhance the overall coherence of your review | Completed: This has been changed to: many patients felt that they lacked control in the decision-making regarding their treatments. The theme of Power, language and communication covers this, but as you rightly suggest, the statement should be worded to fit with the findings. |
|  |  |  |
| R2 |  |  |
| Introduction |  |  |
| 29 | This captures the essence of the background for the systematic review. | Thank you! |
| 30 | I suggest that the details of the 4 stages of the silences framework should be reserved for the methods section. In the background, these stages should only be raised as a conceptual and theoretical matter (with references) rather than a methodological description. | This is now in the methods section and has been reduced to: The Silences Framework^15^ was used as an overarching framework for the whole review. We used 4 stages of the Silences Framework to gain an understanding of what silences currently exist for Black men who have been detained under Mental Health legislation: Stage 1. Working in silences: a systematic literature review to provide a base from which we begin to draw out what is unknown or unsaid about race, ethnicity, and compulsory detention. Stage 2. Hearing silences: reflexive spaces dedicated to listening and hearing individual experiences. Stage 3. Voicing the silences: the analysis of the findings was continuous and cyclical. Initial findings were presented with attendees afforded the opportunity to give feedback.  Stage 4. Working with ‘silences’: Researchers reflect on the potential impact of the findings, and what steps can be taken to achieve the goal or aim of the project.  The four stages are cyclical and each stage is to be considered several times as necessary throughout the life of the research. Through adopting a Silences Framework15 and a truly authentic empowerment model of co-producing the research with people with lived experience, (Black people with experience of detention under mental health legislation or caring for somebody who is, or has been detained), and those with experience of being a professional in these spaces, we collectively and continually questioned what was missing from the research. This authenticity was based on a racialised lived experience that was embedded this into the research from preproduction to coproduction. |
| 31 | Please include the research question and aim/objectives of the review into the prose of the background rather than presenting them in bullets as they currently stand. This will make the background flow gently to its end rather than end abruptly as it has. | Completed - Amended as suggested. |
| Methods |  |  |
| 32 | Please provide a reference for critical realism. | Completed - Reference added |
| 33 | “We conducted a title and abstract search, and along with removing the papers from the additional exclusion criteria, we reduced the papers to 636”. Did the authors mean title and abstract screening? | Completed changed to screening. |
| 34 | “After the full paper review, 72 papers matched the streamlined inclusion criteria”. I thought this should read 32 papers and then when you add the later 2, you have 34. Authors should address this and amend as necessary. | Completed: This was a mistake and has now been amended to 34. |
| 35 | I cannot find table 1 and it was difficult to assess the extent of data summarised on that table. | I'm not sure why this was not accessible to you. |
| 36 | “The silences explored in this paper are just three out of many subthemes born from the workshops held throughout the process…”. On what basis were these three silences chosen as the key issues to be raised in this paper? | A sentence added: The silences explored in this paper are the three most prolific themes arranged from a list of 50 subthemes. |
| 37 | This raises the question of how the thematic analysis was approached in order to generate themes and sub-themes. It is important to understand how the authors randomly chose 50 papers from the second round of searching when in the end only 34 met inclusion for the systematic review? It would imply that some of the 50 papers did not make it into the systematic review but had been used to develop the themes used to interrogate the data for the systematic review as well as the silences attached to them. | Completed: This was changed to: 'The first workshop for the systematic review was held in November 2021, where a coding framework was built utilising the papers from the full paper search' |
| 38 | Additionally, the thematic areas addressed in the narrative synthesis of the systematic review and the attached silences did not entirely follow the main themes identified from the table on themes shown in the supplementary material. It seems there were the final three themes – these should have been included in the table showing themes and sub-themes to make the data presentation clearer. | Completed - the method section has been re-written to clarify this. |
| 39 | While I understand the need of the authors to contextualise their findings and show implication (and might fit more into the recommendations for reflexive thematic analysis), I would suggest that this being a systematic review primarily, the narrative synthesis of the data should be separated from “discussion” of some of the narrated findings so that there will be no repetitions as such. Currently, some aspects of the results would fit better into the discussion as they are not part of the narrative synthesis. In some cases (e.g. “culture, spirituality and religion”), the authors went as far as proffering recommendations for mental health services. This should be reserved for the discussion as it is “slowing down” the narrative in the results section. | The integration of the lived experience voice is incredibly important to the Silences Framework and is a key part of this paper. We do not wish to change this. We have added two sentences to make this clearer in the results section: We believe that this approach helps in understanding what is not being said in current research, and it is important that each theme has a response by people with lived experience. The exploration of the themes is therefore set out as information from the existing literature, as analysed by the full team, with a discussion of the silences that people with lived experience feel is missing. |
| 40 | I am not sure there is a need for two PRISMA flowcharts in the paper. The latter flowchart for the more recent search may be moved to supplementary material to avoid confusion. I will let the editor decide on this. | Completed - changed to one diagram. |
| Discussion |  |  |
| 41 | The discussion should be amended by first discussing the findings from the systematic review and then the silences. This clarity will help the authors to situate their work in the existing literature as expected and also highlight the important gaps left by the silences and what they mean. Currently, some of the findings are not compared with previous literature and are mainly presented as advocacy points. The recommendations/implications raised will then make more meaning that way. | We discussed this point, and our authors with lived experience (in particular) felt that it was important to set out the paper in this way. We did not want to create a systematic review section that people may read and ignore the silences. By putting them side by side, we hope that people will read them together. We have tightened up some of the wording to make them more compatible. We have also addressed this further through adding: Co-producing a systematic review including people with lived experience is a novel approach, and there is currently no framework that determines how this is conducted or written for publication. Therefore, we have written openly about the way our research was conducted and have included as much explanation as possible in the hope that moving forward, a framework for co-producing research may be designed. |
| 42 | The limitations have not been rigorously discussed yet. While they have been raised descriptively, it is important to show how attempts were made to mitigate them. For example, why did the authors proceed to make recommendations from sources that did not feature in the systematic review? Was there a compelling reason to do so – e.g. they provided a good conceptual coverage, they were based on large datasets, sources are scare, etc. | Completed: We have removed the recommendations that were not directly related to findings from the review, we will explore these in more detail in subsequent papers. |
| References |  |  |
| 43 | Inconsistencies noted in the referencing style. Please ensure they conform to the journal style. | Completed |
