# Supplementary material for: A co-produced review of the experiences of Black male detention under mental health legislation: Challenging discrimination in psychiatry using The Silences Framework
Source: PLOS Ment Health. 2025 Apr 9;2(4):e0000041. doi: 10.1371/journal.pmen.0000041 (PMC12798175; doi:10.1371/journal.pmen.0000041)
Supplement: Attachment — Submitted filename: Response to reviewer comments 2.docx [file pmen.0000041.s006.docx]

| **Comment** | **Response** |
| --- | --- |
| 1. Please recast “However, data shows that over double the amount of Black people with mental ill health die in custody than any other demographic…”to “…double the number or proportion of Black people…” | completed |
| 2. Please change “After duplications were removed…” to “After duplicates were removed…”. This is mainly a question of style or convention related to the process. | completed |
| 3. Please place MMAT in brackets. | completed |
| 4. Please recast “We held a total of four workshops in various guises.” to “We held a total of four workshops.” The use of the word “guises” could potentially confuse the reader into thinking that there was some covertness to the workshops or a level of concealment, on the part of the authors, regarding their real intentions for organising the workshops. This did not seem to be the case to me. | completed |
| 5. I would invite the authors to revisit the silences in ‘culture, spirituality and religion’ with regard to the following claim in the paper: “Those with lived experience on the project explained that spiritual practices and beliefs that exist outside of Judeo-Christian religions are often misunderstood and pathologized in mental health services; behaviour. Behaviour or beliefs that do not align with Western concepts of spirituality become a part of the individual’s mental health diagnosis or evidence of a mental illness entirely, which strips them of their autonomy by attributing a choice and a belief that could ground them to their mental state”. Were there instances in which Judeo-Christian beliefs themselves were contextually viewed as pathological by mental health services? This might provide a more nuanced or balanced understanding of the silences. | An example has been added, however, this is not a personal one by people with lived experience, but one from one of the mental health nurses on the team. Personal examples shared from people with lived experiences could be identifiable. |
| 6. In the discussion segment, keep the references to the most recent or most influential in relation to the argument being proposed to avoid making the discussion look like the narrative synthesis all over again. For instance, the discussion around social capital has 16 references. | I think it's quite important to keep them in as we are reiterating how this particular point is very well known, and has been for some years. It does look a bit ridiculous, but that's because it is! |
| 7. It would be helpful for the authors to situate their recommendations in the broader literature so that they could appeal to existing evidence base where possible/available. | Completed, we have added several references throughout the discussion and updated statistics in the introduction. I also noted an error in the numbering of the references so I have rectified this throughout. |
| 8. Could the authors take a more critical approach to discussing the limitations? Lack of generalizability is key and should not be readily minimised. Limitation in relation to the quality of some of the sources e.g. grey literature that were (understandably) not subjected to quality appraisal should be briefly addressed. The predominance of the global North in the papers included (no papers/reviews from the global South really) ought to be discussed as this speaks to generalizability and perhaps global health inequalities between regions of the world (with the implication for sustainable development). | Text has been added to address these issues. |
